# B-cell receptor signaling activity identifies patients with mantle cell lymphoma at higher risk of progression

**DOI:** 10.1038/s41598-024-55728-9

**Published:** 2024-03-19

**Authors:** Simona Gambino, Francesca Maria Quaglia, Marilisa Galasso, Chiara Cavallini, Roberto Chignola, Ornella Lovato, Luca Giacobazzi, Simone Caligola, Annalisa Adamo, Santosh Putta, Antonino Aparo, Isacco Ferrarini, Stefano Ugel, Rosalba Giugno, Massimo Donadelli, Ilaria Dando, Mauro Krampera, Carlo Visco, Maria Teresa Scupoli

**Affiliations:** 1 Department of Engineering for Innovation Medicine, Section of Biomedicine, University of Verona, Verona, Italy; 2 Hematology Unit, Azienda Ospedaliera Universitaria Integrata Verona, Verona, Italy; 3 Department of Neurosciences, Biomedicine and Movement Sciences, Section of Biochemistry, University of Verona, Verona, Italy; 4 Department of Biosystems Science and Engineering, ETH Zurich, Basel, Switzerland; 5 Department of Biotechnology, University of Verona, Verona, Italy; 6 Research Center LURM (Interdepartmental Laboratory of Medical Research), University of Verona, Verona, Italy; 7 Department of Medicine, Section of Immunology, University of Verona, Verona, Italy; 8 Veneto Institute of Oncology IOV-IRCCS, Padua, Italy; 9 BioLegend, Foster City, CA USA; 10 Department of Computer Science, University of Verona, Verona, Italy

**Keywords:** B-cell lymphoma, Phosphorylation

## Abstract

Mantle cell lymphoma (MCL) is an incurable B-cell malignancy characterized by a high clinical variability. Therefore, there is a critical need to define parameters that identify high-risk patients for aggressive disease and therapy resistance. B-cell receptor (BCR) signaling is crucial for MCL initiation and progression and is a target for therapeutic intervention. We interrogated BCR signaling proteins (SYK, LCK, BTK, PLCγ2, p38, AKT, NF-κB p65, and STAT5) in 30 primary MCL samples using phospho-specific flow cytometry. Anti-IgM modulation induced heterogeneous BCR signaling responses among samples allowing the identification of two clusters with differential responses. The cluster with higher response was associated with shorter progression free survival (PFS) and overall survival (OS). Moreover, higher constitutive AKT activity was predictive of inferior response to the Bruton's tyrosine kinase inhibitor (BTKi) ibrutinib. Time-to-event analyses showed that MCL international prognostic index (MIPI) high-risk category and higher STAT5 response were predictors of shorter PFS and OS whilst MIPI high-risk category and high SYK response predicted shorter OS. In conclusion, we identified BCR signaling properties associated with poor clinical outcome and resistance to ibrutinib, thus highlighting the prognostic and predictive significance of BCR activity and advancing our understanding of signaling heterogeneity underlying clinical behavior of MCL.

## Introduction

Mantle cell lymphoma (MCL) is an incurable B-cell malignancy that constitutes 5–7% of all lymphomas^1–3^. The genetic hallmark of MCL is the t(11;14) translocation that juxtaposes the cyclin D1 gene, *CCND1*, to the immunoglobulin heavy or light chain loci, leading to the overexpression of cyclin D1^4^.

MCL is clinically heterogeneous, with some patients having an indolent disease, not requiring treatment for several years, while others experiencing a highly aggressive disease and a dismal outcome^2,5^. Moreover, there remains substantial clinical heterogeneity among patients requiring treatment, with patients experiencing prolonged remission while others rapidly relapsing after therapy^5^.

During the last decades, different clinical and biological parameters have been shown to be associated with patients’ clinical outcomes in MCL. In addition to the clinical prognostic score Mantle Cell Lymphoma International Prognostic Index (MIPI)^6^, elevated tumor cell proliferation, and blastoid morphology variant, both reflecting a high genetic complexity and instability, together with *TP53* mutations or deletions, are associated with unfavorable outcomes^7,8^. Despite the efforts in identifying markers that can stratify patients according to their risk of relapse and death, there is a critical need for more precise definition of expected response and survival to establish a benchmark for clinical guidelines^9^.

Several lines of evidence support the concept that the B-cell receptor (BCR) activity is fundamental for both the initiation and progression of different types of B-cell malignancies, including MCL^10–12^. BCR consists of antigen-binding transmembrane immunoglobulin (mIg) molecules in complex with Igα/Igβ (CD79a/CD79b) transmembrane heterodimers (α/β). mIg subunits bind antigen, resulting in receptor aggregation, while the α/β subunits transduce intracellular signals^13^. Engagement of the BCR activates several nonreceptor protein tyrosine kinases (PTKs), including spleen tyrosine kinase (SYK) and Bruton’s tyrosine kinase BTK, which initiate the formation of a ‘signalosome’ assembling signaling and adaptor molecules^13^. The signalosome coordinates a complex network of signaling transduction involving multiple pathways that are crucial for regulating B-cell fate decisions as well as the survival and proliferation of MCL cells^14^. Thus, in recent years therapeutic strategies blocking the BCR signaling have been developed to treat MCL and other B-cell malignancies^15–17^.

Ibrutinib is a first-in-class inhibitor of Bruton’s tyrosine kinase (BTK), a key protein on the route of the BCR signaling, which binds covalently to the active site of BTK and inhibits kinase’s enzymatic activity and downstream signaling^18^. In MCL, ibrutinib has significantly improved patients’ long-term outcome and overall response among relapsed-refractory patients^19,20^. Despite the remarkable activity of ibrutinib and other BTK inhibitors (BTKi), treatment results are poor in a proportion of patients^19,21–23^ and several mechanisms of intrinsic or adaptive resistance to these selective inhibitors, including activation of compensatory pathways and acquisition of mutations, have been described^24–26^. Thereby, identifying mechanisms of resistance/relapse to BTKi therapy is critically challenging.

In this study, we used phospho-specific flow cytometry to interrogate BCR signaling in primary MCL cell samples. We found evidence that higher signaling response to BCR stimulation identifies patients with inferior survival. Moreover, we showed that constitutive activation of AKT signaling pathway is associated with patients’ resistance to ibrutinib.

## Results

### BCR phosphorylation profiles in primary MCL cells

To functionally characterize the BCR signaling in MCL, the phosphorylation status of nine proteins downstream of the BCR signaling, namely SYK, LCK, BTK, PLCγ2, p38, ERK1/2, AKT, NF-κB p65 and STAT5, were analyzed at the single-cell level in PBMC samples collected from 30 MCL patients at diagnosis or prior to therapy (Table S1) using phospho-specific flow cytometry combined with fluorescent cell barcoding (Figure S1). Phosphorylation of the BCR signaling proteins was measured in the basal (unmodulated) condition and under modulation of the BCR with anti-IgG, anti-IgD, anti-IgM antibodies, or a combination of them (anti-Igs). Circulating B cells from ten healthy donors (HDs) were analyzed as controls (see Supplementary Information, Figure S2 for the study workflow).

The basal activation of BCR phosphoproteins varied across MCL samples particularly for pBTK, pPLCγ2, and pSTAT5 (σ^2^ ≥ 0.1; Figure S3a, b). Although we could not detect differences in the average phosphoprotein levels between MCL and HD samples for each analyzed protein (Figure S3b), variance was higher in MCL than HD samples for pBTK (σ^2^ = 0.14 in MCL *versus* 0.03 in HDs) and pPLCγ2 (σ^2^ = 0.21 in MCL *versus* 0.12 in HDs) whereas comparable variances were calculated for pSTAT5 between MCL and HD samples (σ^2^ = 0.10 in MCL *versus* 0.08 in HDs; Figure S3b).

Most analyzed MCL cell samples expressed surface IgM and IgD while five samples expressed low levels of surface IgG in > 10% of the cells (Table S3; Figure S4). A heatmap of phosphoprotein responses to BCR crosslinking across MCL samples revealed that stimulation with anti-IgM or anti-Igs induced clear-cut responses of BCR proteins, whereas no response was induced by stimulation with anti-IgG or anti-IgD antibodies (Figure S5a). The finding that response to anti-Igs mainly retraced that to anti-IgM alone suggested that anti-Ig-response was driven by crosslinking of BCR with anti-IgM. Therefore, we focused on functional BCR characterization under the anti-IgM-modulated condition. Crosslinking of the BCR with anti-IgM induced significant increased phosphorylation of all the analyzed signaling proteins, except for STAT5 (Figure S5b SYK: *P* < 0.0001; LCK: *P* < 0.0001; BTK: *P* < 0.0001; PLCγ2: *P* < 0.0001; p38: *P* = 0.0005; ERK 1/2: *P* < 0.0001; AKT: *P* < 0.0001; NF-κB: *P* = 0.016). Phosphorylation levels in the anti-IgM-modulated condition across MCL samples were highly variable for pSYK, pBTK, and pPLCγ2 (σ^2^ ≥ 0.1, Figure S5b). Moreover, responses to BCR stimulation showed higher variances in MCL than HD samples for pSYK (σ^2^ = 0.11 in MCL *versus* 0.00 in HD), pBTK (σ^2^ = 0.23 in MCL *versus* 0.04 in HD), and pPLCγ2 (σ^2^ = 0.24 in MCL *versus* 0.02 in HD). Comparison of anti-IgM average responses between MCL and HD cell samples revealed lower p38 and NF-κB p65 responses in MCL than HD samples (Figure S5c; *P* = 0.0036).

To investigate whether the interpatient variability could be due to heterogeneous surface expression levels of IgM, we considered the membrane expression level of IgM in relationship with BCR activation, measured as pBTK response to anti-IgM stimulation. We detected a significant association between the extent of BCR activation and the level of surface IgM expression (Figure S6; *P* = 0.0401), thus confirming previous data^11^.

Overall, these data demonstrate that response to anti-IgM stimulation, which mimics the BCR engagement in the tumor microenvironment, induces activation of the BCR downstream signaling, which is heterogeneous among MCL patients’ samples.

### BCR signaling profiles distinguish subsets of MCL patients

**Figure 1:**
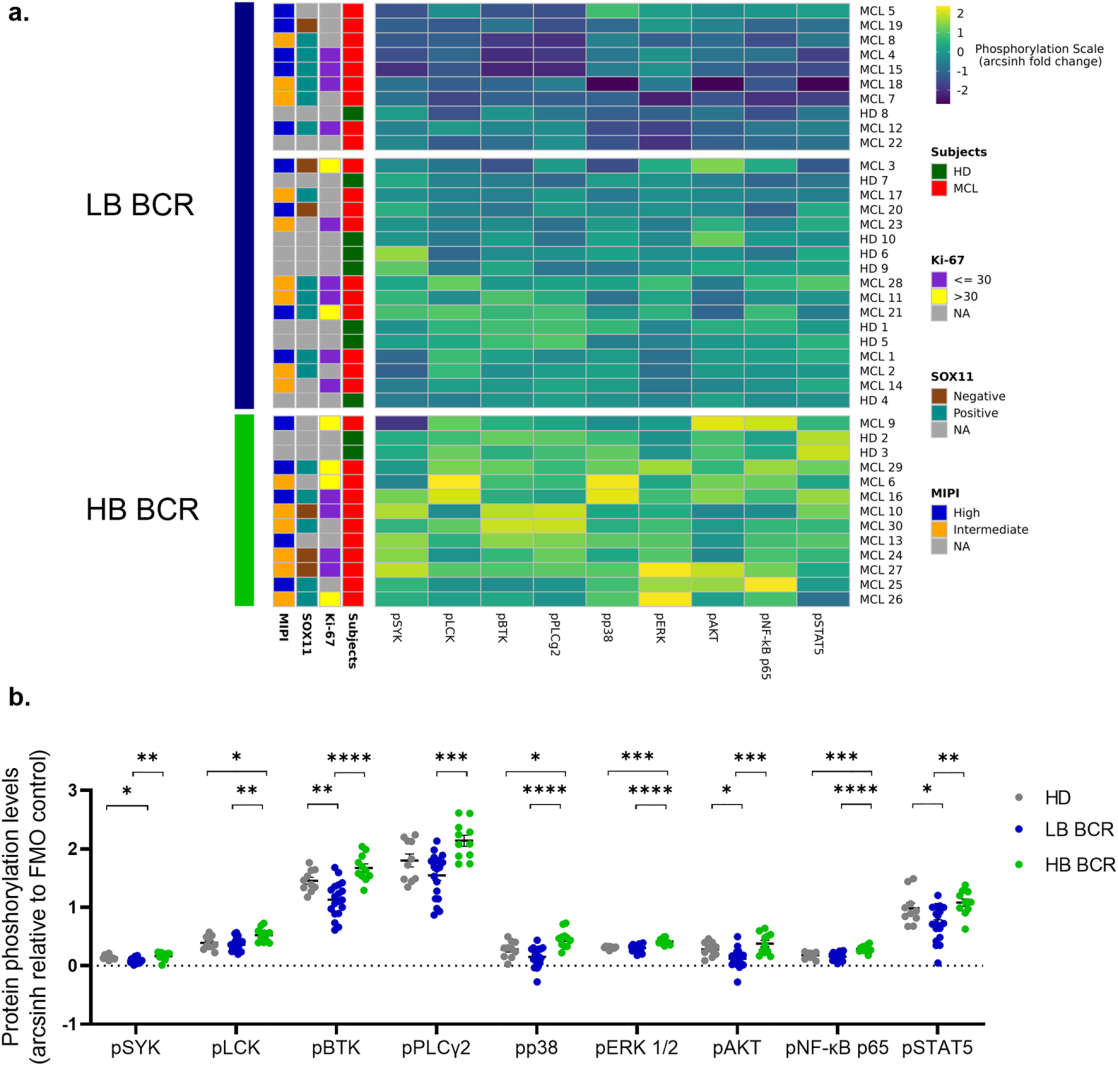
Clustering analysis of basal BCR-protein phosphorylation within MCL and HD samples. (**a**) Unsupervised hierarchical analysis (HCA) of the basal activation status of the BCR phosphoproteins within MCL and HD samples. Data are represented as pseudocolor map corresponding to the arcsinh fold change relative to fluorescence minus one (FMO) control. Each column represents a single phosphoprotein while each row represents a sample (MCL; n = 30; HD; n = 10). Rows were clustered using Euclidian distance and Ward linkage method. Data were z-normalized. (**b**) Comparison of constitutive BCR phosphoprotein activation levels between MCL samples with low basal (LB) BCR (MCL; n = 19), high basal (HB) BCR (MCL; n = 11), and healthy-donor samples (HD; n = 10). MCL groups were obtained after unsupervised HCA analysis. BCR protein phosphorylation status was measured as arcsinh fold change relative to FMO control. Comparison was performed using the one-way ANOVA test. *: *P* < 0.05; **: *P* < 0.01; ***: *P* < 0.001; ****: *P* < 0.0001. Data were reported as mean + SEM.

To assess whether heterogeneity in BCR signaling profiles could allow stratification of MCL patients, data of basal phosphorylation status were subjected to unsupervised hierarchical clustering analysis (HCA) within the MCL and HD cell samples. Unsupervised HCA of phosphoproteins in the basal condition identified two separate clusters. The first cluster comprised samples with higher intrinsic BCR signaling [high basal (HB) BCR] whereas the second one included samples exhibiting a lower basal BCR signaling [low basal (LB) BCR] (Fig. 1a). The latter cluster could be further divided into two clusters characterized by an intermediate and lower basal BCR signaling, respectively (Fig. 1a). Although no difference was observed in the average phosphoprotein levels between MCL and HD samples (Fig. 1b), each analyzed signaling protein was constitutively more phosphorylated in samples from the HB BCR cluster compared with samples from the LB BCR cluster (Fig. 1b; SYK: *P* = 0.0014; LCK: *P* = 0.0015; BTK: *P* < 0.0001; PLCγ2: *P* = 0.0003; p38: *P* < 0.0001; ERK 1/2: *P* < 0.0001; AKT: *P* = 0.0003; NF-κB p65: *P* < 0.0001; STAT5: *P* = 0.0032). Moreover, pLCK, pp38, pERK1/2, and pNF-κB p65 levels were higher in samples from the HB BCR cluster when compared with HD samples (Fig. 1b; *P* = 0.027, *P* = 0.031, *P* = 0.001, *P* = 0.0005, respectively).

**Figure 2:**
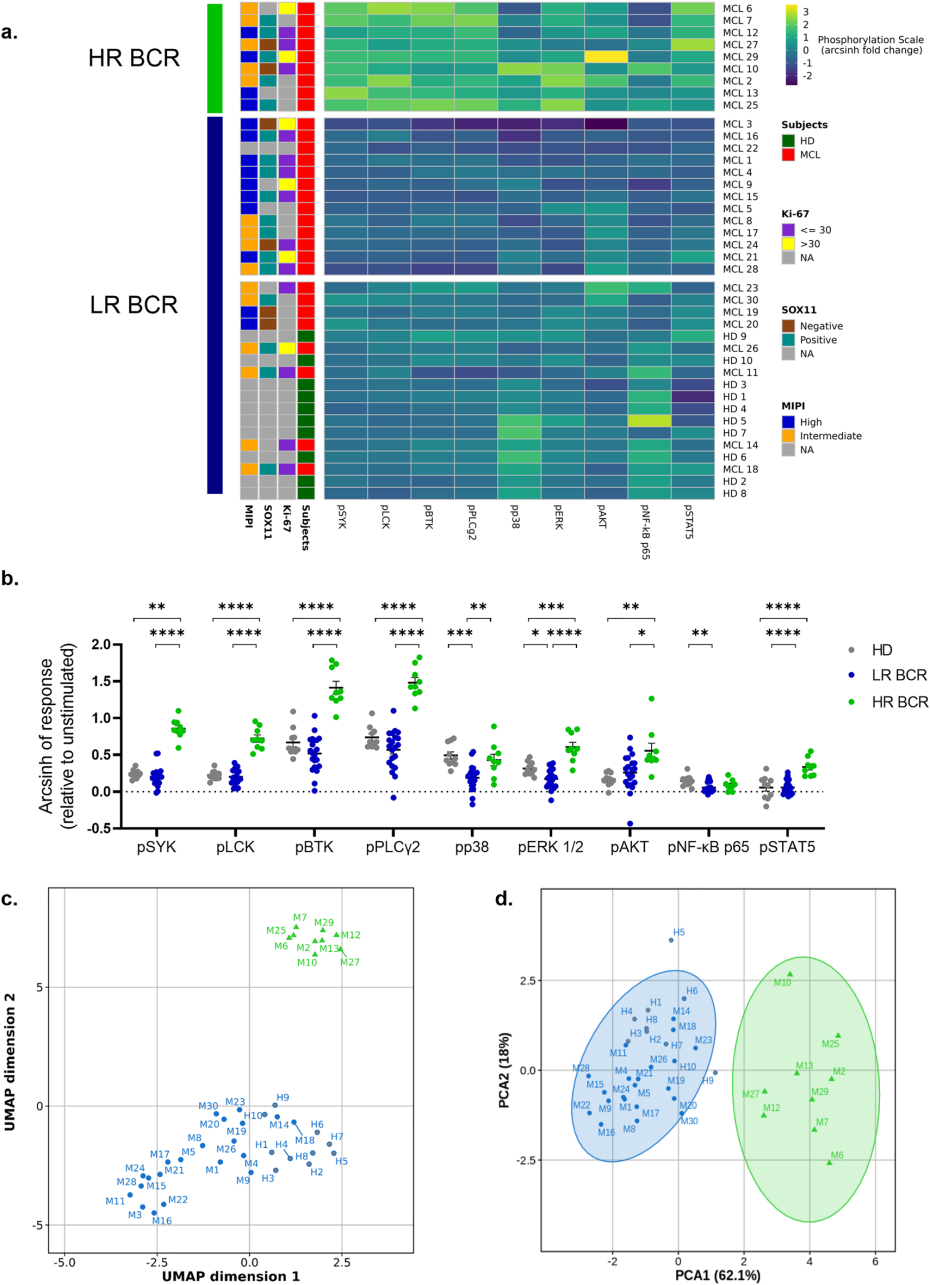
Clustering analyses of anti-IgM-modulated BCR-protein phosphorylation within MCL patients and HD samples. (**a**) Unsupervised hierarchical clustering analysis (HCA) of BCR signaling responses to anti-IgM stimulation within the MCL and HD cell samples. Phosphorylation responses were calculated as arcsinh fold change relative to the basal (unmodulated) condition and represented as pseudocolor map. Each column represents a single phosphoprotein while each row represents a sample (MCL; n = 30; HD; n = 10). Rows were clustered using Euclidian distance and Ward linkage method. Data were z-normalized. (**b**) Comparison of responses to anti-IgM modulation of BCR phosphoproteins in high responder (HR) BCR MCL (n = 9), low responder (LR) BCR MCL (n = 21) and healthy-donor samples (HD; n = 10). Groups were obtained after unsupervised HCA analysis. Phosphorylation levels were calculated as arcsinh fold change relative to the basal (unmodulated) condition. Comparison was performed using the one-way ANOVA test. *: *P* < 0.05; **: *P* < 0.01; ***: *P* < 0.001. Data were reported as mean + SEM. (**c.**) Uniform manifold approximation and projection (UMAP) analysis of MCL and HD samples. In green: HR BCR MCL; in blue: LR BCR MCL; in gray: HD. (**d**) Principal component analysis (PCA) within MCL and HD samples. In green: HR BCR MCL; in blue: LR BCR MCL; in gray: HD.

Unsupervised HCA of BCR responsiveness to anti-IgM modulation within the MCL and HD cell samples also identified two main clusters of MCL patients. The first cluster included samples with greater signaling response to BCR engagement [high responder (HR) BCR] while the second one comprised samples with lower signaling response [low responder (LR) BCR]. LR BCR cluster was further divided into two groups characterized by intermediate and lower responses to BCR, respectively (Fig. 2a). Interestingly, the cluster with an intermediate BCR responsiveness comprised all analyzed HD samples (Fig. 2a). These data were corroborated by comparison of single phosphoprotein data between samples from HR and LR BCR clusters and HD samples. Phosphorylation response of SYK, LCK, BTK, PLCγ2, p38, ERK1/2, AKT and STAT5 to BCR engagement was significantly higher in HR BCR cluster samples compared with LR BCR samples (SYK: *P* < 0.0001; LCK: *P* < 0.0001; BTK: *P* < 0.0001; PLCγ2: *P* < 0.0001; p38: *P* = 0.004; ERK 1/2: *P* < 0.0001; AKT: *P* = 0.029; STAT5: *P* < 0.0001). Moreover, HR BCR samples exhibited a higher BCR response in comparison with HD samples for SYK, LCK, BTK, PLCγ2, ERK1/2, AKT, and STAT5 (Fig. 2b *P* = 0.008, *P* < 0.0001, *P* < 0.0001, *P* < 0.0001, *P* = 0.0001, *P* = 0.002, *P* < 0.0001, respectively). Comparison of BCR response data between LR-BCR and HD samples showed that pp38 and pNF-κB p65 was significantly lower in LR BCR MCL samples (Fig. 2b; *P* = 0.013, *P* = 0.006, respectively). To confirm the results of HCA, we carried out further analyses using two unrelated methods, the nonlinear uniform manifold approximation and projection (UMAP) and the linear principal component analysis (PCA). Both UMAP and PCA within the MCL and HD cell samples identified the same main clusters (HR and LR BCR) determined by HCA, although subclusters within the LR BCR cluster exhibited a partial overlapping (Fig. 2c, d).

### Association of BCR signaling profiles with clinical outcome

We considered the clusters defined by BCR signaling, both under the basal condition and as response to anti-IgM stimulation, in relation to biological and clinical parameters. Fisher’s test showed no association between the BCR-based classification and age at diagnosis, LDH levels, white blood cell (WBC) count, forms/morphological variants, Ki-67, SOX11, MIPI categories (Supplementary Information, Table S6, S7).

**Figure 3:**
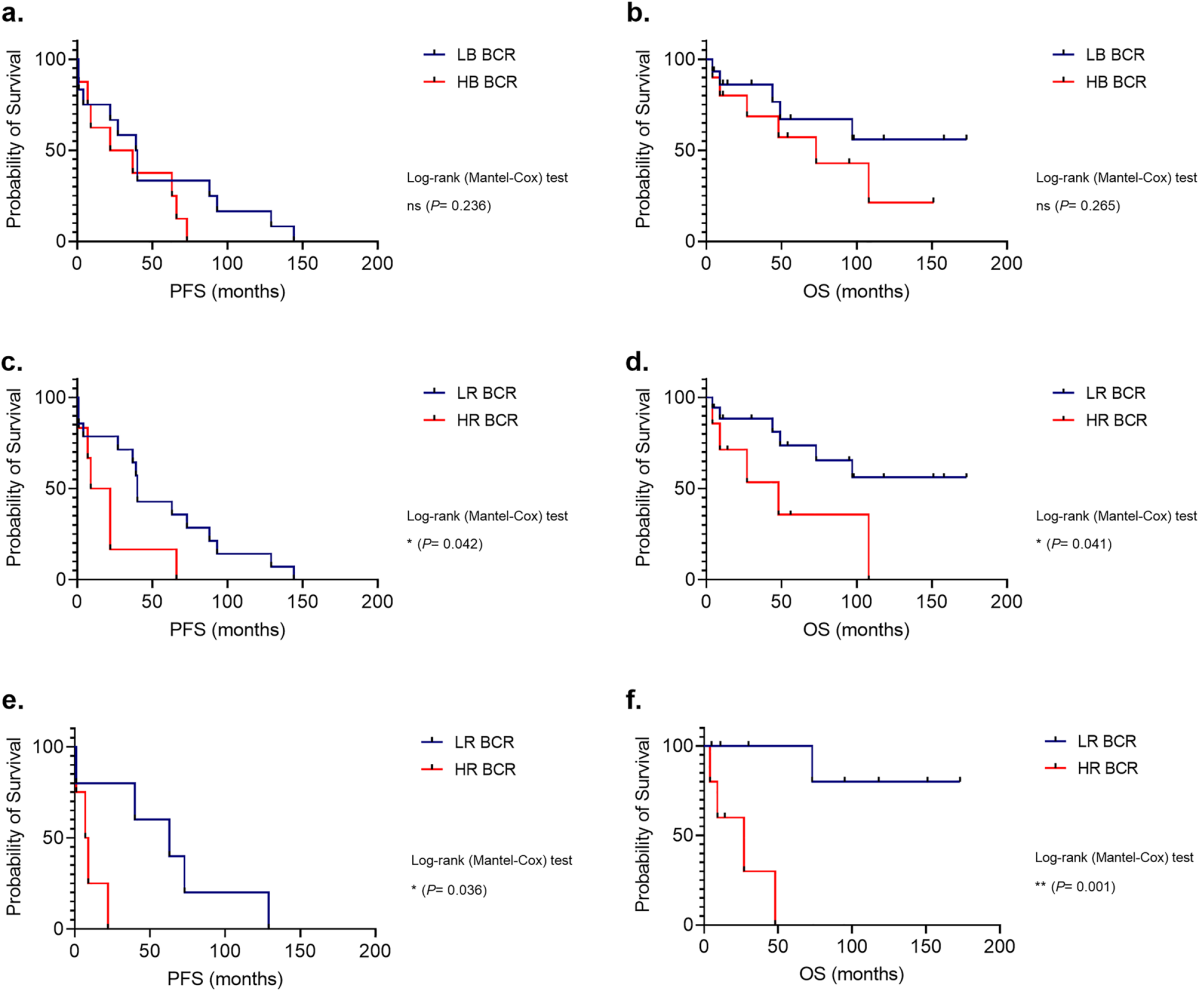
Association of the BCR signaling-based clusters with clinical behavior. Kaplan–Meier curves of progression-free survival (PFS; n = 20) (**a**) and overall survival (OS; n = 25) (**b**) expressed in months for the two MCL clusters defined by BCR basal signaling, i.e., high basal (HB) BCR and low basal (LB) BCR MCL. Kaplan–Meier curves of PFS (n = 20) (**c**) and OS (n = 25) (**d**) expressed in months for the two clusters defined by BCR signaling responsiveness to anti-IgM, i.e., high responder (HR) BCR and low responder (LR) BCR MCL. Kaplan–Meier curves of PFS (n = 9) (**e**) and OS (n = 14) (**f**) expressed in months for the two clusters defined by BCR signaling responsiveness to anti-IgM, i.e., HR BCR and LR BCR within the subset of samples collected at diagnosis. *P* values are from the log-rank test.

Next, we examined the relationship between BCR signaling profiles and clinical outcome. Patient clustering based on constitutive BCR signaling did not show significant association with PFS or OS (Fig. 3a, b). Contrarily, patients grouped in the HR BCR cluster experienced significantly shorter PFS and OS than patients within the LR BCR cluster (median PFS: 15 *versus* 40 months, respectively, log-rank test *P* = 0.042; median OS: 27 *versus* 52 months, respectively, log-rank test *P* = 0.041; Fig. 3c, d). This observation was confirmed when we restricted the analysis to samples collected from patients at lymphoma first diagnosis (median PFS: 8 *versus* 63 months, respectively, log-rank test *P* = 0.036, n = 9; median OS: 14 *versus* 30 months, respectively, log-rank test *P* = 0.001, n = 14; Fig. 3e, f).

### Univariate and bivariate analysis of association of BCR signaling phosphoprotein responses with clinical outcome

The clustering results showed that BCR signaling response profiles contained relevant prognostic information. Therefore, to assess the impact of BCR phosphoprotein activation on MCL patients’ outcome we investigated time-to-event modeling for PFS and OS using both BCR signaling data in the anti-IgM-modulated condition as continuous variables and the available known prognostic parameters (i.e., age, LDH, WBC count, forms/morphological variants, Ki-67, SOX11, MIPI categories). Univariate time-to-event analysis identified increased age at diagnosis and MIPI high-risk category as significant predictors of shorter PFS (LR χ^2^ test *P* = 5·10^–6^ for age at diagnosis; *P* = 2·10^–4^ for MIPI high-risk category; Table 1) and shorter OS (LR χ^2^ test *P* = 7·10^–3^ for age at diagnosis; *P* = 1·10^–3^ for MIPI high risk category; Table 2).

**Table 1:** Selected Cox proportional hazards model for PFS (n = 20).

Variables	βs	SE	*FP*	LR	*P*	Harrell's C
Age at diagnosis	0.213	0.052	4.9·10^–5^	20.87	5·10^–6^	0.862
MIPI high	2.571	0.80	1.4·10^–3^	14.22	2·10^–4^	0.735
Anti-IgM→pSTAT5	3.6466	1.65	2.7·10^–2^	18.84	8·10^–5^	0.77
MIPI high	3.1583	0.89	3.9·10^–4^			

βs: indicates beta coefficients; SE: standard error of estimated coefficients; *FP*: feature-specific p-value; LR: likelihood ratio; *P*: global p-value; Harrell's C: concordance index to evaluate the predictive performance of a survival model.

**Table 2:** Selected Cox proportional hazards model for OS (n = 25).

Variables	βs	SE	*FP*	LR	*P*	Harrell's C
Age at diagnosis	0.12	0.049	1.4·10^–2^	7.31	7·10^–3^	0.747
MIPI high	2.748	1.09	1.2·10^–2^	10.2	1·10^–3^	0.735
Anti-IgM→pSYK	1.771	0.87	4.1·10^–2^	14.1	9·10^–4^	0.806
MIPI high	2.9029	1.11	9.2·10^–3^			
Anti-IgM→pSTAT5	4.593	1.19	1.6·10^–2^	15.83	4·10^–4^	0.812
MIPI high	3.470	1.91	3.5·10^–3^			

βs: indicates beta coefficients; SE: standard error of estimated coefficients; *FP*: feature-specific p-value; LR: likelihood ratio; *P*: global p-value; Harrell's C: concordance index to evaluate the predictive performance of a survival model.

In bivariate time-to-event analysis, both the global p-value *P* and the p-values of individual components (feature-specific p-value, *FP*) indicated significant associations in the model. For the prediction of shorter PFS, a higher response of pSTAT5 to anti-IgM modulation (anti-IgM → pSTAT5; *FP* = 2.7·10^–2^) and MIPI high-risk (*FP* = 3.9·10^–4^) were identified as independent and significant predictors. The overall significance of the model was confirmed by the LR χ^2^ test (*P* = 8·10^–5^; Table 1).Similarly, in the analysis of OS, two models were found to be significant. One model combined a higher response of pSYK to anti-IgM modulation (anti-IgM → pSYK; *FP* = 4.1·10^–2^) with the MIPI high-risk category (*FP* = 9.2·10^–3^). The other model combined a higher response of pSTAT5 to anti-IgM modulation (anti-IgM → pSTAT5; *FP* = 1.6·10^–2^) with the MIPI high-risk (*FP* = 3.5·10^–3^). The LR χ^2^ test confirmed the significance of these models (*P* = 9·10^–4^ and *P* = 4·10^–4^, respectively; Table 2).

In summary, both the global p-value and the feature specific p-values demonstrated the significance of the model in predicting shorter PFS and OS, providing evidence of the observed associations.

### Association of BCR signaling data with response to ibrutinib

**Figure 4:**
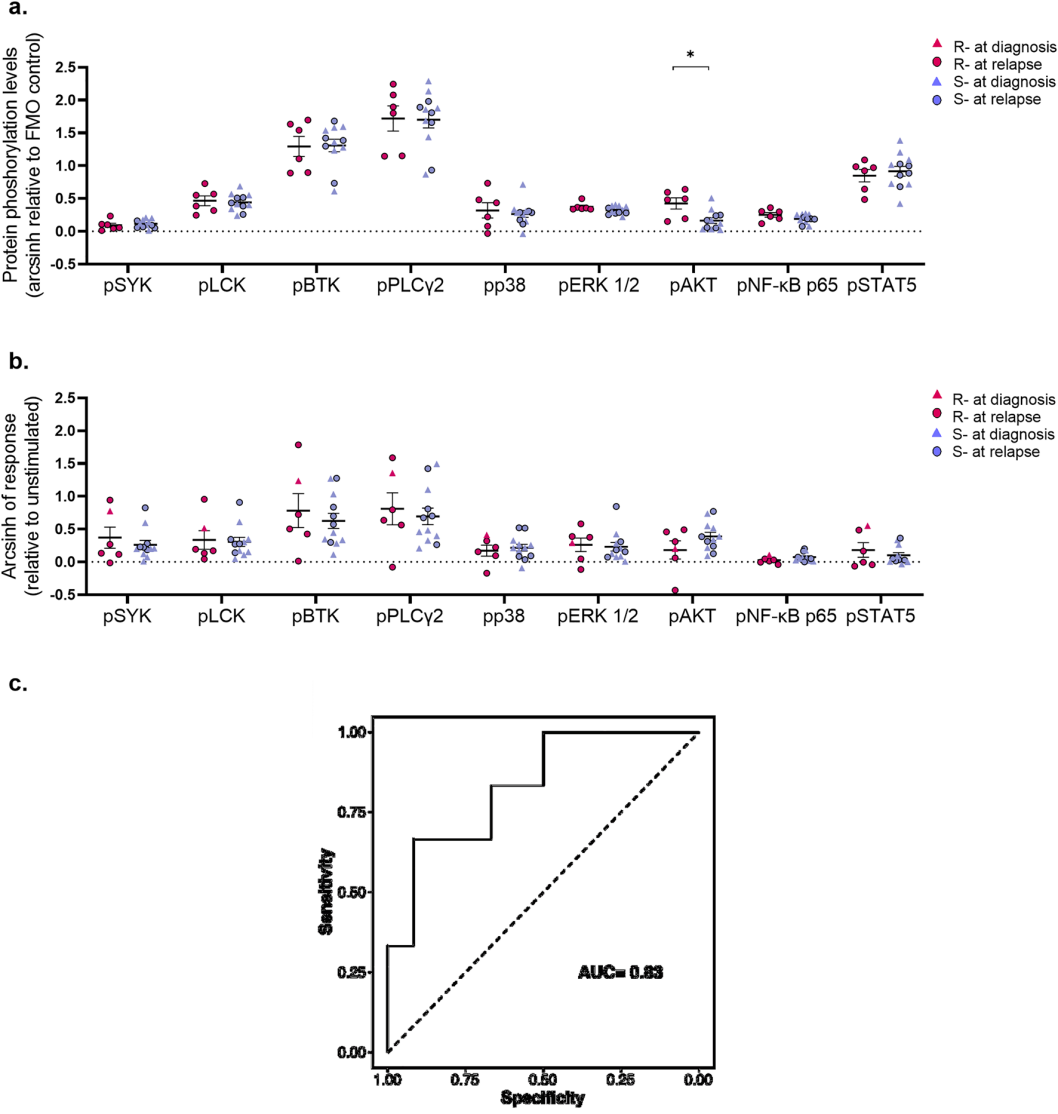
Association of the BCR signaling level with the response to ibrutinib therapy. (**a**) Comparison of constitutive phosphorylation status for each signaling protein between samples from refractory (R; n = 6; at diagnosis n = 1, at relapse n = 5) and sensitive (S; n = 12; at diagnosis n = 7, at relapse n = 5) MCL patients to ibrutinib therapy. BCR protein phosphorylation was measured as arcsinh fold change relative to fluorescence minus one (FMO) control. (**b**) Comparison of responses to BCR modulation for each signaling protein between samples from refractory (R; n = 6; at diagnosis n = 1, at relapse n = 5) and sensitive (S; n = 12; at diagnosis n = 7, at relapse n = 5) MCL patients to ibrutinib therapy. BCR responsiveness was calculated referring to the basal (unmodulated) condition. Comparison was performed using the Mann–Whitney test. *: *P* < 0.05. Data were reported as mean + SEM. (**c**) Receiver operating characteristic curve (ROC) showing the classification performance of basal pAKT levels on the response to ibrutinib therapy as modeled by logistic regression.

We analyzed BCR signaling profiles in relation to the response to ibrutinib. Patients that were resistant to ibrutinib exhibited a significantly higher level of pAKT in the basal condition compared with patients responsive to the drug (Fig. 4a; *P* = 0.023). In contrast, no association was observed between responses of BCR phosphoproteins to anti-IgM modulation and response to ibrutinib (Fig. 4b). Association of higher constitutive pAKT levels and ibrutinib resistance of MCL patients was confirmed by a logistic regression analysis, which correctly predicted 83% of the data with higher levels of basal pAKT associated with a higher probability of being resistant to ibrutinib therapy (Table 3; Fig. 4c).

**Table 3:** Ibrutinib response data logistic regression as the function of basal pAKT levels (n = 18).

Coefficients	Estimate	SE	*FP*	Deviance analysis	
				*LR*	*P*
β0	− 2.899	1.223	1.8·10^–2^	15.707	7.3·10^–3^
β1	7.746	3.481	2.6·10^–2^		

The response to therapy with ibrutinib was coded as 1 = resistant and 0 = sensitive; SE: standard error of estimated coefficients. *FP*: feature-specific p-value; LR: likelihood ratio; *P*: global p-value.

## Discussion

In this study, we characterize functional profiles of BCR signaling in primary MCL cells. The key advance of this study is the identification of B-cell signaling profiles that are associated with poor survival and ibrutinib resistance in patients with MCL.

The first result from this study documents a constitutively active BCR signaling in MCL, specifically for BTK and PLCγ2 that exhibit elevated phosphorylation levels. BTK and PLCγ2 were constitutively active also in peripheral blood B cells from healthy donors, presumably for their fundamental role in survival of healthy and malignant B cells^27^. Importantly, we describe a higher constitutive BCR signaling in a subset of MCL patients’ samples, with all the analyzed phosphoproteins being constitutively more activated compared with the remaining MCL samples. These findings highlight the pivotal role of BCR signaling activity in survival and proliferation of MCL cells and in disease pathogenesis^10,11,14^. However, the basal levels of BCR phosphoproteins do not contain prognostic information since they are not associated with either known prognostic parameters or clinical outcomes. These data are in contrast with previous findings from Myklebust et al.^11^, who showed a significant association between basal activation of the BCR downstream signaling and OS. This finding could be partially due to the different tissues that were used in the two studies, peripheral blood in our study and prevalently lymph nodes in the Myklebust’s one^11^. Indeed, one possibility is that MCL cells derived from lymph nodes could maintain ex vivo the BCR activity induced by tumor microenvironment in vivo, thus being more similar to the stimulated condition of MCL cells in our study.

Although constitutive BCR signaling appears to be not prognostically relevant, herein we show that it may contain important predictive information. Indeed, higher constitutive activation of AKT identifies a subgroup of patients who are resistant to ibrutinib treatment. AKT is activated by PI3K indirectly through the production of PIP3 (phosphatidyl-inositol 3,4,5-trisphosphate) that binds AKT, facilitating its activation by other kinases^28^. The PI3K/AKT pathway plays a key role in regulating cell function, growth, and proliferation in various cancers and is unusually active in the pathogenesis of MCL^29–31^. In aggressive MCL, a point mutation in the gene coding for BTK leads to enhanced activation of BTK-mediated signaling and to activation of AKT circuitry^24,25^. Also, inactivation of phosphatase and tensin homolog (PTEN) confers constitutive activation of AKT, which may promote chemoresistance^32^. Interestingly, a recent study shows that the PI3K/AKT signaling confers tumor microenvironment–driven ibrutinib resistance in MCL^33^. Therefore, the finding that ibrutinib-resistant cells exhibit constitutive activation of the AKT pathway is consistent with a role for AKT in resistance mechanisms and overall support the combination use of BTK and PI3K/AKT pathway inhibitors in MCL. Further investigations are needed to elucidate molecular mechanisms underlying AKT constitutive activation and conferring ibrutinib resistance in a subset of MCL. Importantly, integrating signaling data with genomic mutational information could be of crucial relevance to identify patients who are more likely to relapse while on ibrutinib therapy and offer alternative treatment options.

BCR modulation with anti-IgM induces a clear-cut and heterogeneous response of the BCR downstream phosphoproteins in terms of activation status, comparing to the unmodulated control. The strength of BCR signaling response correlates with the levels of cell surface IgM expression, thus confirming previous data^11^. Although we do not observe any difference in average response to BCR modulation between MCL and HD samples, interpatient response variability—specifically for pBTK, pPLCγ2, and pSTAT5—is higher within MCL than HD samples, confirming that MCL is a disease with heterogeneous biological features and BCR signaling^11,34^. Moreover, we document a higher BCR responsiveness to IgM modulation in a subset of MCL patients’ samples, with all the analyzed phosphoproteins (but pNF-κB p65) responding more actively compared with the remaining MCL samples. Remarkably, we show that higher BCR signaling responsiveness identifies a subgroup of patients with shorter PFS and OS. Interestingly, association between increased BCR signaling responsiveness and faster disease progression is maintained for PFS and improved for OS in the subset of samples collected from patients at diagnosis, who would benefit primarily from an improved risk stratification.

Consistently, in a bivariate time-to-event analysis, the model combining higher response of pSYK to anti-IgM with MIPI high-risk category shows to be significantly associated with shorter OS. Moreover, a higher response of pSTAT5 to anti-IgM combined with MIPI high-risk category is an independent predictor of shorter PFS and OS in bivariate time-to-event analyses. These results point to pSYK and pSTAT5 as relevant signaling nodes on the route of BCR signaling that can capture the behavior of other components of the pathway, supporting the direct functional interrogation of diseased cells. Together with BTK, SYK plays a pivotal role in regulation of survival, proliferation, and homing of malignant B cells. Upon activation, SYK recruits BTK and adaptor molecules triggering downstream events that lead to propagation of AKT, MAPK, and NF-κB signaling and upregulation of the Bcl-2 family proteins^35^. SYK is overexpressed and hyperactivated in MCL cells^36^ and may be a target of therapy^37–39^. The role of STAT5 is poorly defined in MCL. However, a recent study suggests that activated STAT5 could inhibit the expression of tumor suppressor cytokine signaling 2 (SOCS), thus promoting MCL pathogenesis^40^. Moreover, elevated constitutive levels of pSTAT5 have been shown to be associated with poor outcome in MCL^11^. Overall, these data show that responsiveness profiles of signaling proteins to BCR stimulation, rather than the basal levels of protein phosphorylation, are correlated with disease progression in MCL. These findings agree with our previous data in CLL^41,42^ and with the concept that exposing cancer cell signaling to potentiating inputs, rather than relying upon the basal levels of protein phosphorylation alone, can identify unique cancer signaling profiles that correlate with disease outcome^43^.

In conclusion, this study identifies BCR signaling profiles that are associated with poor clinical outcome and resistance to the BTKi ibrutinib, thus advancing our understanding of signaling heterogeneity underlying clinical behavior of MCL. Future challenges are to use BCR signaling data integrated with genetic signature to predict patient’s clinical behavior and drug response for a more personalized treatment approach.

## Methods

### Patients and healthy donors

Peripheral blood mononuclear cell (PBMC) samples from 30 MCL patients and from 10 age-matched healthy donors (HDs) were collected and cryopreserved at the Hematology Unit, Azienda Ospedaliera Universitaria Integrata (AOUI) in Verona (Italy) on approval from the local Ethics Committee (Comitato Etico per per la Sperimentazione Clinica delle Province di Verona e Rovigo, AOUI). In accordance with the Declaration of Helsinki, all patients and HDs provided written informed consent to the use of their biological material for research purposes. Nineteen peripheral blood samples were collected at diagnosis prior to any treatment and 11 peripheral blood samples were collected at relapse, at least six months after the last therapy and prior any other. Clinical and biological features of MCL samples are summarized in Supplementary Information, Table S1. Details on patient clinical characteristics and inclusion criteria are described in Supplementary Information.

### Cell preparation and detection of surface immunoglobulins

PBMCs from MCL patients and HDs were isolated by Ficoll hypaque centrifugation (Lymphoprep; Nicomed, NO, EU) and stored in liquid nitrogen. Upon thawing, sample viability was assessed using 7-amino-actinomycin (7-AAD) dye (BD Biosciences, San Jose, CA) by flow cytometry (FACS Canto II, Becton Dickinson, FranklinLakes, NJ). Only samples with viability > 85% were further processed. After thawing, MCL PBMCs were stained with fluorochrome-conjugated antibodies for immunophenotype analysis of surface immunoglobulin (Ig) expression. Approximately 10,000 events were acquired on a BD LSR Fortessa X20 (Becton Dickinson). List and characteristics of anti-surface marker antibodies are detailed in Supplementary Information, Table S2. Data on MCL cell surface expression of Igs are reported in Supplementary Information, Table S3.

### Cell treatments and phospho-specific flow cytometry

Upon thawing, cells were rested at 2.4·10^6^/ml for 2 h at 37 °C in RPMI 1640 GlutaMAX (Thermo Fisher Scientific, Waltham, MA) supplemented with 10% fetal bovine serum and 1% penicillin/streptomycin. After 2-h rest at 37 °C, PBMCs were treated for 10 min at 37 °C with goat F(ab’)_2_ anti-human IgG; goat F(ab’)_2_ anti-human IgD; goat F(ab’)_2_ anti-human IgM (all from SouthernBiotech, Birmingham, AL) at 20 μg/ml each, a mix of them, or left unmodulated, as detailed in Supplementary Information.

Phospho-specific flow cytometry was performed as previously described^41,44^. Briefly, after stimulation PBMCs were fixed with paraformaldehyde (PFA) 2% (Thermo Fisher Scientific) at room temperature for 10 min and then permeabilized with ice-cold 75% methanol at − 20 °C for 30 min. After rehydrating with PBS, the different conditions were differentially labeled with 1:4 serial dilution of Pacific blue Succinimidyl ester fluorescent dye (Thermo Fisher Scientific) at 4 °C for 30 min. Specifically, 100 μg/ml was used for the anti-Ig-mix condition; 25 μg/ml for the anti-IgM condition; 6.25 μg/ml for the anti-IgD condition; 1.56 μg/ml for the anti-IgG condition; vehicle (DMSO) for the unstimulated condition. Then, “barcoded” cells were mixed and stained with fluorochrome-conjugated antibodies at 4 °C for 30 min, as previously describedx^45,46^. List, and characteristics of antibodies used are detailed in Supplementary Information, Table S4. The antibody panel used for phosphoprotein detection is described in Supplementary Information, Table S5. Approximately 10,000 events of each condition (50,000 total gated events) were acquired on BD LSR Fortessa X20 (Becton Dickinson). Flow cytometry data processing and analysis are described in Supplementary Information. A representative gating strategy is showed in Figure S1. To measure phosphorylation statuses of signaling proteins, we used the inverse hyperbolic sine (arcsinh) fold change. Median fluorescence intensity (MFI) signals were divided by the scale argument (set at 150) and transformed in arcsinh. To express the magnitude of protein phosphorylation status in the basal or modulated condition, arcsinh values were normalized with respect to the reference control, i.e., fluorescence minus one (FMO). Responsiveness of cells modulated under specific stimulation was calculated as change relative to the unmodulated condition^11^.

### Statistical analysis

Progression-free survival (PFS) was calculated from initiation date of upfront therapy to the date of relapse/progression or death from any cause. Overall survival (OS) was calculated from date of upfront therapy to death or last follow-up. Patients who did not receive any treatment were excluded from survival analysis. PFS and OS curves estimated using the Kaplan–Meier method for the respective groups of patients were compared using the log-rank (Mantel-Cox) test. Univariate and bivariate models for PFS and OS were generated using Cox proportional hazards regression. Resistance to ibrutinib was defined as failure to achieve at least a partial response to the drug according to Lugano classification of lymphoid malignancies^47^.

Fisher’s exact test, unpaired student t test, Mann–Whitney test, one-way ANOVA corrected for multiple comparisons, Pearson correlation were used as appropriate. Normal (Gaussian) distribution of data was tested with the D'Agostino-Pearson normality test. Differences were considered statistically significant for *P* ≤ 0.05. Variability was calculated as sigma squared (variance, σ^2^). The unsupervised hierarchical cluster analysis (HCA) was calculated using Euclidean distances as metric and the Ward linkage as method of clustering. The unsupervised HCA, uniform manifold approximation and projection (UMAP), principal component analysis (PCA), logistic regression modeling, and receiver operating characteristic (ROC) curve were performed using the R software (v. 3.0). Graphing and statistical analyses were performed using GraphPad Prism software (v.7.05; GraphPad Software Inc., La Jolla, CA, USA).

### Ethics approval and consent to participate

MCL samples were collected on approval from the local Ethics Committee (Comitato Etico per per la Sperimentazione Clinica delle Province di Verona e Rovigo, AOUI). In accordance with the Declaration of Helsinki, all patients and HDs provided written informed consent to the use of their biological material for research purposes.

### Supplementary Information

Supplementary Information.

## Data Availability

All data generated or analyzed during this study are included in this published article [and its supplementary file]. Flow cytometry raw data are published in Flow repository at the following link: http://flowrepository.org/id/FR-FCM-Z75F.
