# Supplementary material for: B-cell receptor signaling activity identifies patients with mantle cell lymphoma at higher risk of progression
Source: Sci Rep. 2024 Mar 19;14:6595. doi: 10.1038/s41598-024-55728-9 (PMC10951201; doi:10.1038/s41598-024-55728-9)
Supplement: Supplementary file 1 — Supplementary Information. [file 41598_2024_55728_MOESM1_ESM.pdf]

# B-cell receptor signaling activity identifies patients with mantle cell lymphoma at higher risk of progression

Simona Gambino[1#], Francesca Maria Quaglia[2#], Marilisa Galasso[3], Chiara Cavallini[4], Roberto Chignola[5], Ornella Lovato[6], Luca Giacobazzi[7], Simone Caligola[8], Annalisa Adamo[7], Santosh Putta[9], Antonino Aparo[6], Isacco Ferrarini[1,2], Stefano Ugel[7], Rosalba Giugno[10], Massimo Donadelli[3], Ilaria Dando[3], Mauro Krampera[1,2], Carlo Visco[1,2*] and Maria Teresa Scupoli[3,6*]

[#]SG and FMQ equally contributed to the study
[1]Department of Engineering for Innovation Medicine, Section of Biomedicine of Innovation, Hematology and Bone Marrow Transplant Unit, University of Verona, Verona, Italy
[2]Hematology Unit, Azienda Ospedaliera Universitaria Integrata Verona, Verona, Italy
[3]Department of Neurosciences, Biomedicine and Movement Sciences, University of Verona, Verona, Italy
[4]Department of Biosystems Science and Engineering, ETH Zurich, Basel, Switzerland
[5]Department of Biotechnology, University of Verona, Verona, Italy
[6]Research Center LURM (Interdepartmental Laboratory of Medical Research), University of Verona, Verona, Italy
[7]Department of Medicine, Section of Immunology, University of Verona, Verona, Italy
[8]Veneto Institute of Oncology IOV-IRCCS, Padua, Italy
[9]BioLegend, Foster City, California, US
[10]Department of Computer Science, University of Verona, Verona, Italy.

**Corresponding authors:**

*Maria Teresa Scupoli, PhD, Laboratorio Universitario di Ricerca Medica (LURM), Policlinico G.B. Rossi, P.le L. A. Scuro, 10, 37134 Verona – Italy; Phone: +39-045-812 8425, e-mail: mariateresa.scupoli@univr.it

*Carlo Visco, MD, Divisione di Ematologia, Policlinico G.B. Rossi, P.le L. A. Scuro, 10, 37134 Verona – Italy; Phone: +39-045-812 4420, e-mail: carlo.visco@univr.it

## Supplementary Information

*MCL patient clinical characteristics and inclusion criteria*

MCL patient inclusion criteria were the histologically documented diagnosis of MCL as defined in the 2016 edition of the WHO classification[1], the availability of cryopreserved PBMC samples, the availability of clinical annotation. Exclusion criteria were unavailability of cryopreserved PBMC samples, any histology other than MCL. Characteristics of patients are summarized in Table S1. The study cohort consisted of 30 MCL patients with epidemiological and clinical characteristics consistent with the general MCL population. Patients were diagnosed between 2010 and 2022, and were followed for relapse, re-treatment, and death with data cutoff September 15$^{th}$ 2022; all events were validated by review of the medical record. Patients were treated according to local practice at the discretion of physician, either with intensive or non-intensive therapeutic regimens. Eighteen samples were collected from patients before starting ibrutinib treatment as second line, comprising n=12 sensitive patients and n=6 refractory patients to ibrutinib. Patients who did not receive any treatment (n=5) were excluded from overall survival (OS) analysis. Moreover, patients who did not progress after first line treatment (n=5) were excluded from progression free survival (PFS) analysis. Hence, as regards samples collected at lymphoma first diagnosis (n=19), n=14 patients were included in the OS analysis and n=9 patients were included in the PFS analysis.

*Cell treatments and phospho-specific flow cytometry*

Upon thawing, cells were rested at 2.4 x $10^6$/ml for 2 hours at 37°C in RPMI 1640 GlutaMAX (Thermo Fisher Scientific, Waltham, MA) supplemented with 10% fetal bovine serum and 1% penicillin/streptomycin. Then, MCL samples were treated at 37°C for 10 minutes with anti-IgG, anti-IgD, anti-IgM, or a mix of them (anti-Igs), as specified in Methods. The ten-minute time point for anti-Ig modulation was chosen based on kinetic analyses and because induced representative, although not necessarily maximal, phosphorylation responses of all BCR proteins. The chosen anti-Ig concentration was consistent with those used in previous studies.[2,3]

*Flow cytometry data processing and analysis*

Immunophenotype data were processed and analyzed using Flow Jo software (v10; TreeStar, Ashland, OR) while phospho-specific flow cytometry data were analyzed using Ryvett (Qognit, Biolegend, Foster City, CA) or Cytobank (Beckman Coulter, Brea, CA) software. Instrument stability during acquisition was checked through time parameter. Debris, doublets and dead cells were excluded based on FSC-A/SSC-A, FSC-A/FSC-H and cPARP positivity. T cells and macrophages were excluded based on CD3 and CD14 positivity, respectively. MCL-B cells were identified based on CD19/CD5 coexpression. Eventually, each stimulation condition was identified based on Pacific blue dye fluorescent intensity. A representative gating strategy is shown in Figure S1.

To measure phosphorylation statuses of signaling proteins, we used the inverse hyperbolic sine (arcsinh) fold change. Arcsinh transformation combines linear transformation for values close to zero and logarithmic one for larger values, normalizing raw data by the scale argument. Therefore, the transformed data will have similar distribution to the non-normalized data that were acquired using bi-exponential scale, better reflecting the actual extent of fluorescence shift. [4–6]

## Tables

### Table S1. Clinical and biological features of MCL patients

| | |
|---|---|
| **Number of cases** | 30 |
| **Median age, year (range)** | 76 (47-87) |
| **[a]Median LDH levels (U/L) (range)** | 193 (125-546) |
| **[b]Median WBC count ($10^9$/L)** | 9 (3-206) |
| **Forms/Morphological variants** | |
| **Classical** | 22 |
| **Blastoid/pleomorphic** | 3 |
| **Leukemic non-nodal** | 4 |
| **ND** | 1 |
| **Median proliferation index (Ki-67 %) (range)** | 20 (3-80) |
| **[c]SOX11** | |
| **Positive** | 17 |
| **Negative** | 6 |
| **ND** | 7 |
| **[d]MIPI** | |
| **Low** | 1 |
| **Intermediate** | 14 |
| **High** | 14 |
| **ND** | 1 |
| **Median overall survival, months (range)** | 47 (1-176) |
| **Median progression free survival, months (range)** | 38 (1-144) |
| **Specimen timepoint** | |
| **Diagnosis** | 19 |
| **Relapse, pre-therapy** | 11 |

ND: not determined

[a]LDH: lactate dehydrogenase

[b]WBC: white blood cells

[c]SOX11 immunohistochemistry: >30% positive, ≤30% negative

[d]MIPI: mantle cell lymphoma international prognostic index

**Table S2. Antibodies used for surface immunoglobulin detection**

| Antibody | Fluorochrome | Manufacturer | Clone |
|---|---|---|---|
| **Mouse Anti-human IgG** | FITC | Becton Dickinson | G18-145 |
| **Mouse Anti-human IgD** | PE | Becton Dickinson | IA6-2 |
| **Mouse Anti-human IgM** | APC | Becton Dickinson | G20-127 |
| **Mouse anti-human CD19** | BV786 | BD | SJ25C1 |
| **Mouse anti-human CD5** | PerCP-Cy5.5 | Thermo Fisher | L17F12 |

**Table S3. Expression of surface immunoglobulins in MCL samples**

| Patient ID | CD19/CD5 positive cells (%) | IgG positive cells (%) | IgD positive cells (%) | IgM positive cells (%) |
|---|---|---|---|---|
| **MCL 1** | 70.6 | 0.016 | 96.7 | 100 |
| **MCL 2** | 7.0 | 2.47 | 57.2 | 93.7 |
| **MCL 3** | 0.3 | 8.78 | 33.1 | 75 |
| **MCL 4** | 7.7 | 1.1 | 91.7 | 99.4 |
| **MCL 5** | 86.2 | 8.66 | 99.7 | 99.9 |
| **MCL 6** | 1.2 | 1.99 | 70.5 | 82.9 |
| **MCL 7** | 24.8 | 0.29 | 49.3 | 98.2 |
| **MCL 8** | 60.4 | 11.3 | 95 | 99.9 |
| **MCL 9** | 0.56 | 3.5 | 27.3 | 100 |
| **MCL 10** | 48.5 | 13.9 | 100 | 69.4 |
| **MCL 11** | 1.4 | 4.19 | 67.4 | 71.6 |
| **MCL 12** | 44.0 | 1.17 | 98.1 | 89 |
| **MCL 13** | 56.1 | 4.09 | 99.5 | 99.3 |
| **MCL 14** | 5.7 | 4.73 | 45.9 | 73 |
| **MCL 15** | 6.8 | 2.15 | 83.3 | 88.3 |
| **MCL 16** | 3.7 | 0.58 | 95.5 | 38.5 |
| **MCL 17** | 63.6 | 0.81 | 98.9 | 41.2 |
| **MCL 18** | 3.8 | 10.7 | 6.94 | 76.3 |
| **MCL 19** | 64.8 | 0.36 | 18.1 | 67.2 |
| **MCL 20** | 82.0 | 3.82 | 98.8 | 55.8 |
| **MCL 21** | 1.5 | 4.41 | 41.9 | 52 |
| **MCL 22** | 2.7 | 0.85 | 88.9 | 4.7 |
| **MCL 23** | 75.0 | 0.65 | 81.9 | 100 |
| **MCL 24** | 66.7 | 13.1 | 99.9 | 94.4 |
| **MCL 25** | 83.2 | 2.82 | 99.8 | 72.8 |
| **MCL 26** | 18.4 | 0.79 | 97.6 | 92.7 |
| **MCL 27** | 71.9 | 14.4 | 99 | 72.5 |
| **MCL 28** | 35.2 | 7.54 | 43.2 | 55.3 |
| **MCL 29** | 68.6 | 1.38 | 97.1 | 96.2 |
| **MCL 30** | 75.1 | 5.9 | 99.6 | 92.7 |

**Table S4. Antibodies used for phospho-specific flow cytometry analyses**

| Antibody | Fluorochrome | Manufacturer | Clone |
|---|---|---|---|
| **Mouse anti-ZAP70 (pY319)/Syk (Y352)** | AF488 | Becton Dickinson | 17A/P-ZAP70 |
| **Mouse anti-ERK1/2 (pT202/pY204)** | AF488 | Becton Dickinson | 20A |
| **Mouse anti-Btk (pY223)/Itk (pY180)** | AF488 | Becton Dickinson | N35-86 |
| **Mouse anti-PLC-γ2 (pY759)** | PE | Becton Dickinson | K86-689.37 |
| **Mouse anti-NF-κB p65 (pS529)** | PE | Becton Dickinson | K10-895.12.50 |
| **Mouse anti-Human Lck (pY505)** | PE | Becton Dickinson | 4/LCK-Y505 |
| **Mouse anti-Stat5 (pY694)** | AF647 | Becton Dickinson | 47/Stat5(pY694) |
| **Mouse anti-p38 MAPK (pT180/pY182)** | AF647 | Becton Dickinson | 36/p38 (pT180/pY182) |
| **Rabbit anti-human AKT (S473)** | AF647 | Cell signaling | 193H12 |
| **Mouse anti-Human CD19** | BV786 | Becton Dickinson | SJ25C1 |
| **Mouse anti-Cleaved PARP (Asp 214)** | AF700 | Becton Dickinson | F21-852 |
| **Mouse anti-CD5** | PerCP-Cy5.5 | Thermo Fisher | L17F12 |
| **Mouse anti-CD3** | APCCy7 | Biolegend | UCHT1 |
| **Mouse anti-CD14** | APCCy7 | Biolegend | M5E2 |

**Table S5. Antibody panel used for phosphoprotein detection**

| Tube | AF488 | PE | AF647 | APCCy7 | BV786 | PerCP-Cy5.5 | BV450 | AF700 |
|---|---|---|---|---|---|---|---|---|
| **Tube 1** | $pSYK^{Y352}$ | $pPLC\gamma2^{Y759}$ | $pSTAT5^{Y694}$ | CD3+CD14 | CD19 | CD5 | Pacific blue | cPARP |
| **Tube 2** | $pERK1/2^{Y202/Y204}$ | pNF-κB $p65^{S536}$ | $pAKT^{S473}$ | CD3+CD14 | CD19 | CD5 | Pacific blue | cPARP |
| **Tube 3** | $pBTK^{Y223}$ | $pLCK^{Y505}$ | $pp38^{T180/Y182}$ | CD3+CD14 | CD19 | CD5 | Pacific blue | cPARP |
| **FMO AF488** | - | $pLCK^{Y505}$ | $pSTAT5^{Y694}$ | CD3+CD14 | CD19 | CD5 | Pacific blue | cPARP |
| **FMO PE** | $pSYK^{Y352}$ | - | $pAKT^{S473}$ | CD3+CD14 | CD19 | CD5 | Pacific blue | cPARP |
| **FMO AF647** | pERK $1/2^{Y202/Y204}$ | $pPLC\gamma2^{Y759}$ | - | CD3+CD14 | CD19 | CD5 | Pacific blue | cPARP |

**Table S6. Association of basal BCR-based clusters and clinical parameters**

| Parameters | LB BCR | HB BCR | *P* value |
|---|---|---|---|
| **Age at diagnosis** | | | *0.708* |
| <70 | 9 | 4 | |
| ≥70 | 10 | 7 | |
| **LDH (U/L)** | | | *>0.999* |
| ≤225 | 14 | 5 | |
| >225 | 13 | 1 | |
| **WBC ($10^9$/L)** | | | *0.392* |
| ≤4 | 0 | 1 | |
| >4 | 17 | 10 | |
| **Forms/morphology** | | | *0.393* |
| Classic | 13 | 9 | |
| Blastoid/Pleomorphic | 2 | 1 | |
| Leukemic non-nodal | 3 | 1 | |
| **Ki-67 (%)** | | | *0.318* |
| ≤30 | 9 | 4 | |
| >30 | 2 | 4 | |
| **SOX11 (%)** | | | *0.621* |
| ≤30 | 3 | 3 | |
| >30 | 12 | 5 | |
| **MIPI** | | | *>0.999* |
| Low/intermediate | 9 | 6 | |
| High | 9 | 5 | |

**Table S7. Association of anti-IgM-induced BCR-based clusters and clinical parameters**

| Parameters | LR BCR | HR BCR | *P* value |
|---|---|---|---|
| **Age at diagnosis** | | | *0.229* |
| <70 | 11 | 2 | |
| ≥70 | 10 | 7 | |
| **LDH (U/L)** | | | *>0.999* |
| ≤225 | 13 | 6 | |
| >225 | 3 | 1 | |
| **WBC ($10^9$/L)** | | | *0.321* |
| ≤4 | 0 | 1 | |
| >4 | 19 | 8 | |
| **Forms/morphology** | | | *0.825* |
| Classic | 16 | 6 | |
| Blastoid/Pleomorphic | 2 | 1 | |
| Leukemic non-nodal | 2 | 2 | |
| **Ki-67 (%)** | | | *>0.999* |
| ≤30 | 10 | 3 | |
| >30 | 4 | 2 | |
| **SOX11 (%)** | | | *>0.999* |
| ≤30 | 4 | 2 | |
| >30 | 12 | 5 | |
| **MIPI** | | | *>0.999* |
| Low/intermediate | 10 | 5 | |
| High | 10 | 4 | |

## Figures

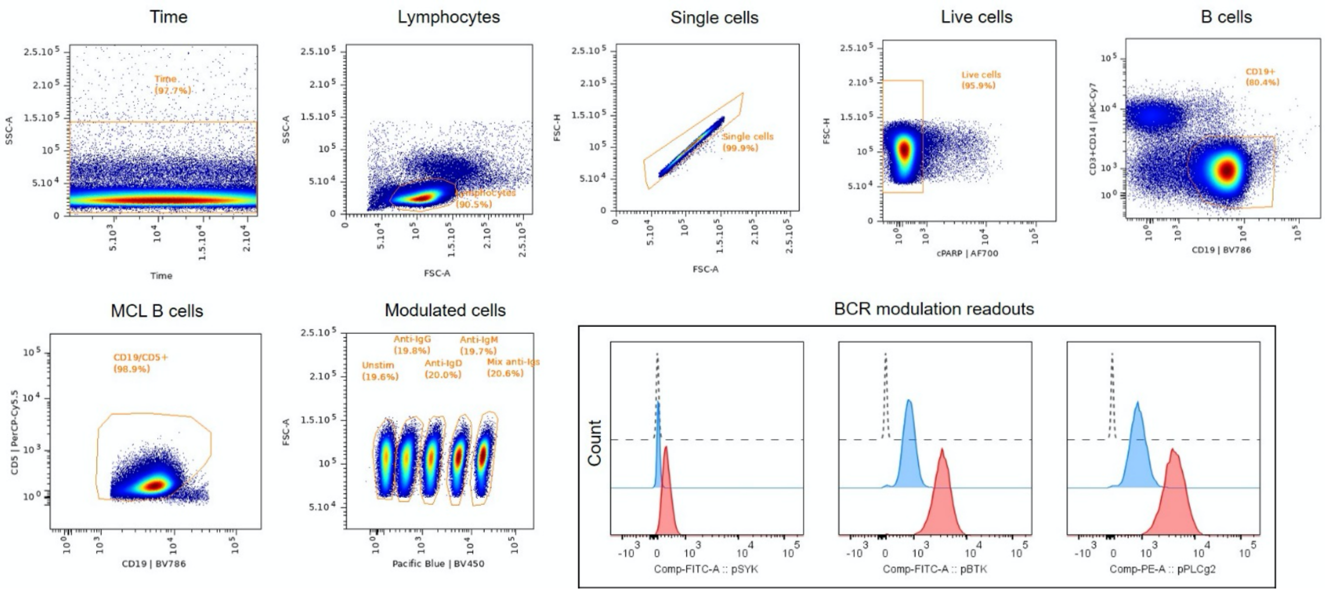


**Figure S1. Representative gating strategy used for BCR phosphoprotein analyses.** Data relative to phospho-specific flow cytometry were analyzed using Ryvett (Qognit, Biolegend) or Cytobank (Beckman Coulter) software. The stability of the instrument during the acquisition was checked through time parameter. Debris, doublets and dead cells were excluded based on FSC-A/SSC-A, FSC-A/FSC-H and cPARP positivity, respectively. T cells and macrophages were excluded based on CD3 and CD14 positivity, respectively. MCL-B cells were identified based on CD19/CD5 coexpression. Eventually, each stimulation condition was identified based on Pacific blue dye fluorescent intensity. Phosphoprotein fluorescence intensity values were expressed referring to the fluorescence minus one (FMO) control.

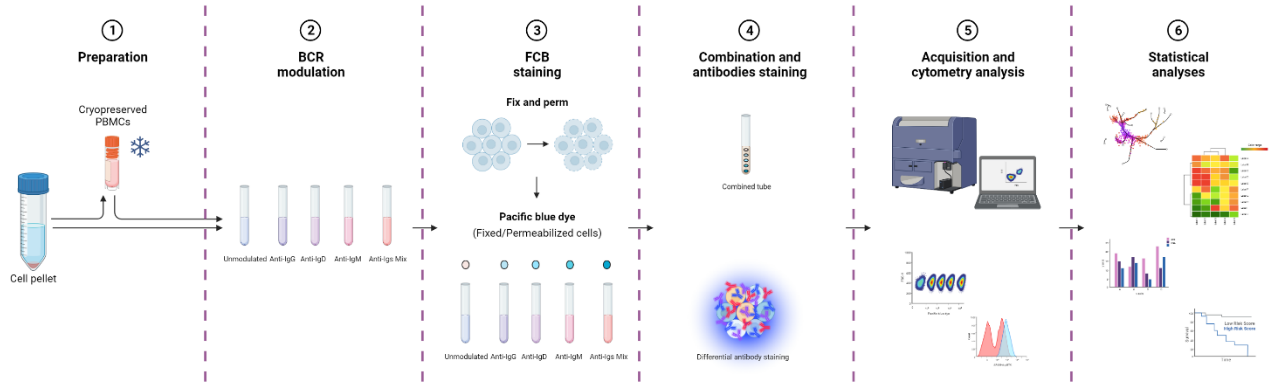


**Figure S2. Experimental workflow for the BCR signaling analysis by phospho-specific flow cytometry in MCL.** MCL patient peripheral blood was collected in tubes with EDTA K2 or EDTA K3 (1,7 or 2,8 mg per ml of blood, respectively). Peripheral blood mononuclear cells (PBMC) were isolated by Ficoll hypaque centrifugation and stored in liquid nitrogen. Upon thawing, sample viability was assessed using 7-amino-actinomycin (7-AAD) dye by flow cytometry. Only samples with viability >85% were further processed. Cells were rested at 2.4 x $10^6$/ml for 2 hours at 37°C and then stimulated at 37°C for 10 minutes with anti-IgG, -IgD, -IgM at 20 $\mu$g/ml each or anti-Igs mix or left unstimulated. After modulation, cells were fixed with 2% paraformaldehyde (PFA) at room temperature for 10 minutes and permeabilized with 75% methanol (MetOH) at -20°C for 30 minutes. After rehydrating the cells by in phosphate-buffered saline (PBS), the different conditions were differentially labeled with 1:4 serial dilution of Pacific blue Succinimidyl ester fluorescent dye at 4°C for 30 minutes. Particularly, 100 $\mu$g/ml was used for the anti-Igs mix condition; 25 $\mu$g/ml for the anti-IgM condition; 6,25 $\mu$g/ml for the anti-IgD condition; 1,56 $\mu$g/ml for the anti-IgG condition; vehicle (DMSO) for the unstimulated condition. "Barcoded" cells were mixed and divided in 6 different tubes for fluorochrome-conjugated antibody staining at 4°C for 30 minutes. Approximately 10.000 events of each condition (50.000 total gated events) were acquired on BD LSR Fortessa X20 (Becton Dickinson).

**a.**

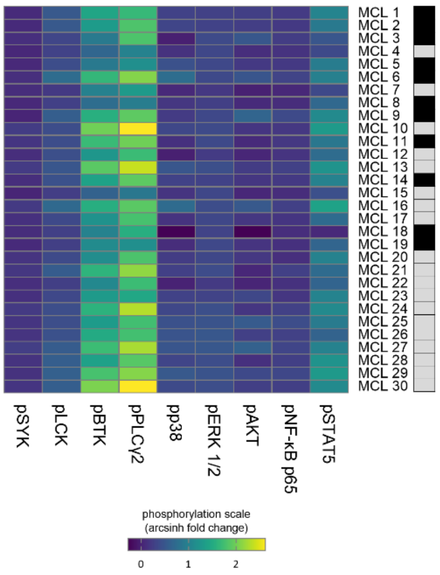


**b.**

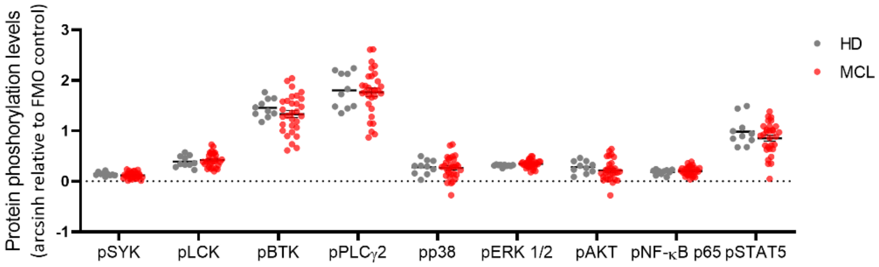


**Figure S3. Basal phosphorylation levels of BCR phosphoproteins in MCL and HD samples.** Samples were thawed and rested for 2 hours at 37°C before analysis. Phosphorylation status of phosphoproteins has been calculated as arcsinh fold change relative to fluorescence minus one (FMO) control. (**a.**) Pseudocolor map of BCR protein constitutive phosphorylation levels in B cells from MCL patients (MCL; n=30). In black: samples at relapse (n=19); in gray: samples at diagnosis (n=11). (**b.**) Comparison of BCR phosphoprotein activation levels in the basal condition between B cells from MCL patients (MCL; n=30) and healthy donors (HD; n=10). Comparison was performed using the Student's t test and data were reported as mean+SEM.

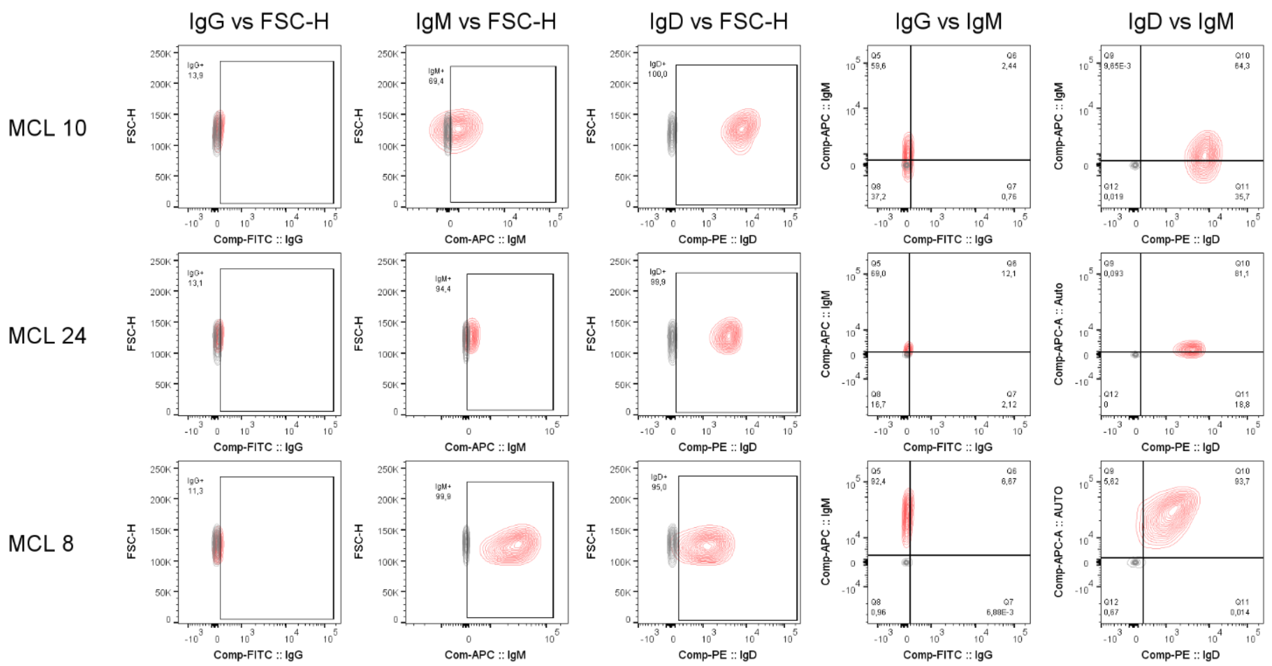


**Figure S4. Representative contour plots of the surface immunoglobulins (Igs) in MCL samples.** Data relative to immunophenotype characterization were analyzed using FlowJo software (Tree Star). Igs expression was quantified on live MCL cells (CD19/CD5+). In gray: autofluorescence negative control; in red: stained sample.

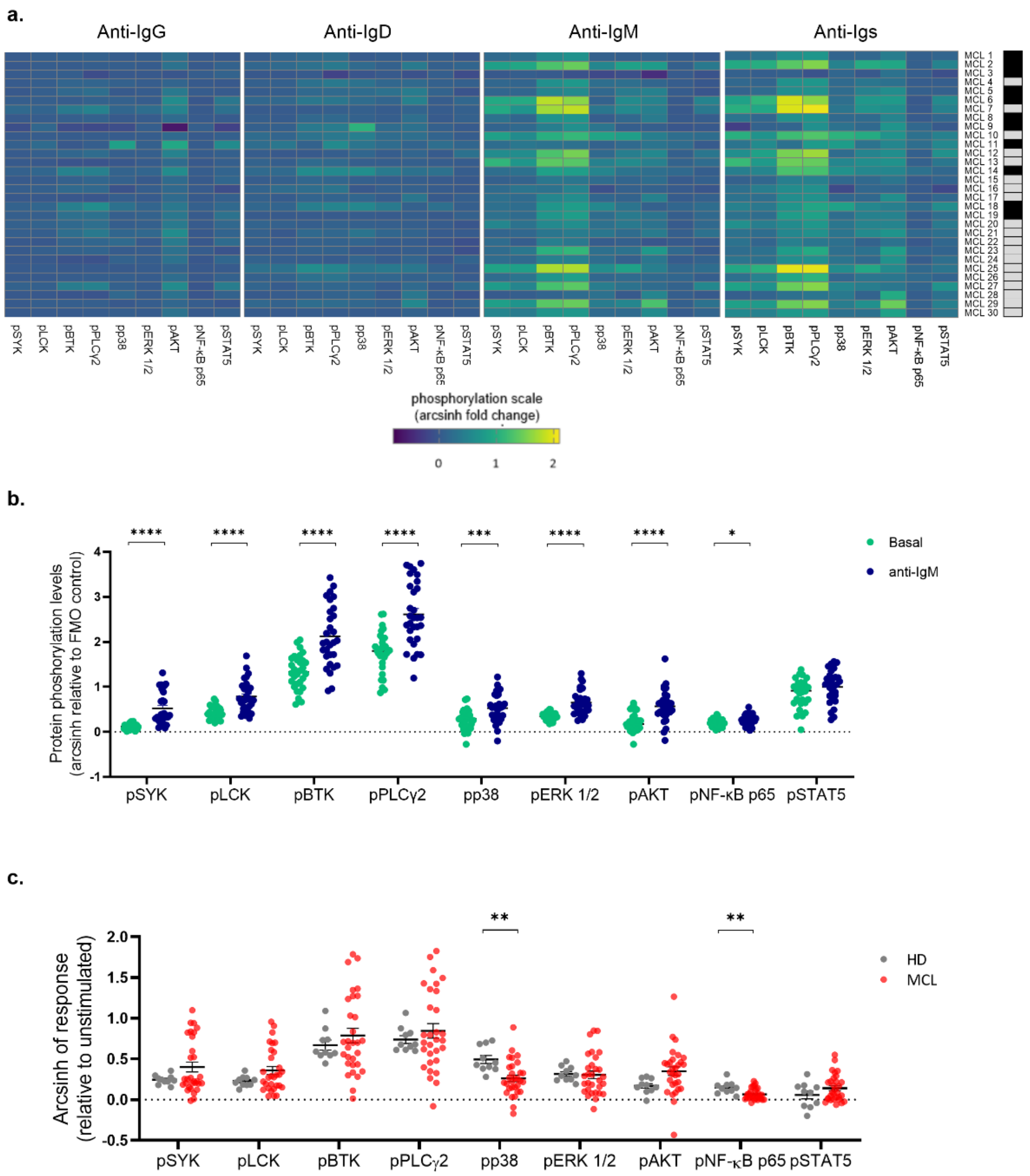


**Figure S5. BCR-induced phosphorylation levels of BCR phosphoproteins in MCL and HD samples.** (**a.**) MCL cell samples were stimulated with 20 μg/ml anti-IgG, -IgD, -IgM, or a mix of them (anti-Igs) at 37°C for 10 minutes and phosphorylation status was calculated as arcsinh fold change relative to fluorescence minus one (FMO) control. Data are represented as pseudocolor map. In black: samples at relapse (n=19); in gray: samples at diagnosis (n=11). (**b.**) Comparison of BCR phosphoprotein activation statuses in the basal and anti-IgM modulated conditions. Phosphorylation level was calculated as arcsinh fold change relative to FMO. (**c.**) Comparison of BCR signaling responses to anti-IgM modulation between B cells from MCL patients (MCL; n=30) and healthy donor samples (HD; n=10). BCR responsiveness was calculated as arcsinh fold change relative to the basal (unmodulated) condition. Comparison of signaling activation was performed using the Student's t test. *: $P<0.05$; **: $P<0.01$; ***: $P<0.001$; ****: $P<0.0001$. Data were reported as mean+SEM.

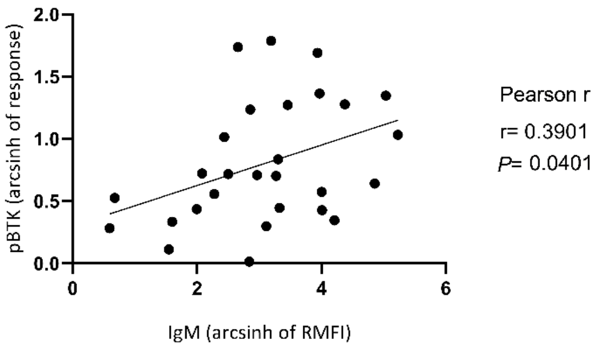


**Figure S6. Association between BCR-induced signaling and IgM surface expression.** Correlation between BTK phosphorylation induced by anti-IgM and surface expression of IgM. BTK phosphorylation response to anti-IgM modulation was calculated referring to the basal (unmodulated) condition. IgM surface expressions was calculated referring to autofluorescence control. The Pearson correlation coefficient is indicated with the probability value for the null hypothesis of no correlation.

## References


1. Swerdlow, S. H. *et al.* The 2016 revision of the World Health Organization classification of lymphoid neoplasms. *Blood* vol. 127 2375–2390 at https://doi.org/10.1182/blood-2016-01-643569 (2016).
2. Cavallini, C. *et al.* Low catalase expression confers redox hypersensitivity and identifies an indolent clinical behavior in CLL. *Blood* **131**, 1942–1954 (2018).
3. Cavallini, C. *et al.* Effects of CD20 antibodies and kinase inhibitors on B-cell receptor signalling and survival of chronic lymphocytic leukaemia cells. *Br. J. Haematol.* **192**, 333–342 (2021).
4. Irish, J. M. *et al.* B-cell signaling networks reveal a negative prognostic human lymphoma cell subset that emerges during tumor progression. *Proc. Natl. Acad. Sci. U. S. A.* **107**, 12747–12754 (2010).
5. Folcarelli, R. *et al.* Transformation of multicolour flow cytometry data with OTflow prevents misleading multivariate analysis results and incorrect immunological conclusions. *Cytom. Part A* **101**, 72–85 (2022).
6. Finak, G., Perez, J. M., Weng, A. & Gottardo, R. Optimizing transformations for automated, high throughput analysis of flow cytometry data. *BMC Bioinformatics* **11**, 1–13 (2010).
